# Efficient Estimation of Mutation Rates during Individual Development by Minimization of Chi-Square

**DOI:** 10.1371/journal.pone.0135398

**Published:** 2015-08-12

**Authors:** Shi-Meng Ai, Jian-Jun Gao, Shu-Qun Liu, Yun-Xin Fu

**Affiliations:** 1 Laboratory for Conservation and Utilization of Bioresources, Yunnan University, Kunming, 650091, China; 2 Department of Applied Mathematics, Yunnan Agricultural University, Kunming, 650201, China; 3 Human Genetics Center and Division of Biostatistics, School of Public Health, The University of Texas Health Science Center, Houston, Texas, 77030, United States of America; Indiana University Bloomington, UNITED STATES

## Abstract

Mutation primarily occurs when cells divide and it is highly desirable to have knowledge of the rate of mutations for each of the cell divisions during individual development. Recently, recessive lethal or nearly lethal mutations which were observed in a large mutation accumulation experiment using *Drosophila melanogaster* suggested that mutation rates vary significantly during the germline development of male *Drosophila melanogaster*. The analysis of the data was based on a combination of the maximum likelihood framework with numerical assistance from a newly developed coalescent algorithm. Although powerful, the likelihood based framework is computationally highly demanding which limited the scope of the inference. This paper presents a new estimation approach by minimizing chi-square statistics which is asymptotically consistent with the maximum likelihood method. When only at most one mutation in a family is considered the minimization of chi-square is simplified to a constrained weighted minimum least square method which can be solved easily by optimization theory. The new methods effectively eliminates the computational bottleneck of the likelihood. Reanalysis of the published *Drosophila melanogaster* mutation data results in similar estimates of mutation rates. The new method is also expected to be applicable to the analysis of mutation data generated by next-generation sequencing technology.

## Introduction

Fundamental to many areas of biology is mutation and the rate at which mutation occurs is one of the central themes in evolutionary biology. Mutation occurs primarily when cells divide. For sexual organisms, there are tens to hundreds of cell divisions during individual development from a fertilized egg to the cells in the tissues of interest. The rates of mutation during individual development is not only of interest to evolutionary biology but also provides crucial information for the study of somatic genetic disease, such as cancers/tumors.

Mutations in the lineage of cells leading to sperm or eggs, known as germline, is most relevant to population genetics and evolutionary biology. For years, there was little knowledge about the detailed pattern of mutation during the germline development, partly due to the difficulty and expense in obtaining sufficient amounts of data for understanding such patterns and also partly due to the lack of appropriate statistical methods for making the required inference. A recent study [1–3] on the recessive lethal or nearly lethal mutations during the germline development of male *Drosophila melanogaster* shed some light on this important area of biology. The experimental data reported in Gao et al. [1,3] and Fu [2] were obtained from a large scale mutation accumulation and screening experiment, the basis for which has been refined over the years [4–6]. In general, the germline development of a male *Drosophila melanogaster* consists of about 36–40 cell divisions, among which the first 14 cell divisions belong to the cleavage stage, the last 5 to spermatogenesis, and those in between to gastrulation and organogenesis. Gao et al. [1,3] and Fu [2] concluded that the mutation rates in the mature young male *Drosophila melanogaster* vary significantly during the germline development. In particular, mutation rate for the first cell division is the highest, followed by those during spermatogenesis. The framework of statistical inference used in Gao et al. [1,3] and Fu [2] is an approximated maximum likelihood approach, in which, the probability of an observed mutation pattern is approximated and the coefficients necessary for the likelihood computation were obtained through computer simulation based on a newly developed coalescent algorithm [1–3,7,8] for the germline lineage of male *Drosophila melanogaster* [9–11].

Although the maximum likelihood based approach advocated by Gao et al. [1,3] and Fu [2] is powerful in principle and flexible in dealing with different levels of complexity in the experimental data, it is computationally demanding. The computational burden results mainly from two components. One is the many coefficients that are required to compute the probability of an observed mutation pattern and that their values need to be estimated using simulated samples. Despite that the newly developed coalescent algorithm [1–3] is very efficient, this process is lengthy and requires substantial resources with the increasing complexity of mutation patterns. The second component contributing to the high demand of computation comes from the high-dimensional search of the maximum likelihood estimates of mutation rates. This aspect of inference is not trivial since the search is a constrained optimization problem and widely used optimization methods, such as the Newton-Raphson method, often fail. As a result, a relatively slow grid search algorithm [1–3] has been used which severely limited the scope of the inference. With sufficient amounts of data, one should be able, in principle, to make inferences about the mutation rate for each cell division during the germline development, but due to the computational burden, Gao et al. [1,3] and Fu [2] were able to examine mutations rates by dividing 36–40 cell divisions into 5–6 intervals with mutation rate assumed to be constant within each interval. For example, Gao et al. [1] divided the 36 cell divisions into 5 intervals: [1,1], [2,2], [3,14], [15,31], and [32,36].

The purpose of this paper is to present a new approach based on chi-square statistics [12] to estimate the mutation rate and to apply the new method to the data reported in Gao et al. [1,3]. When only at most one mutation in a family is considered, the method is equivalent to a constrained weighted least square method which can be easily solved using optimization theory. The new method will be shown to be computationally efficient and sufficiently accurate for a wide range of mutation rates, thus it provides an effective alternative approach which complements the maximum likelihood method. Reanalysis of data in Gao et al. [1,3] using the new method arrives at consistent estimates of mutation rates.

## Theory and Methods

Fundamental to the inference on mutation rates during the individual development is the probability of a mutation pattern in a family. That is, the number of offspring that carry the mutation. The characterization of such probability requires the knowledge of the dynamics of individual development which determines the genealogical properties of a sample of cells within an individual. The detailed exploration of this aspect was made in Gao et al. [1,3] and Fu [2] and this paper will present the components that are pertinent to the formulation of our new approach.

**Fig 1:**
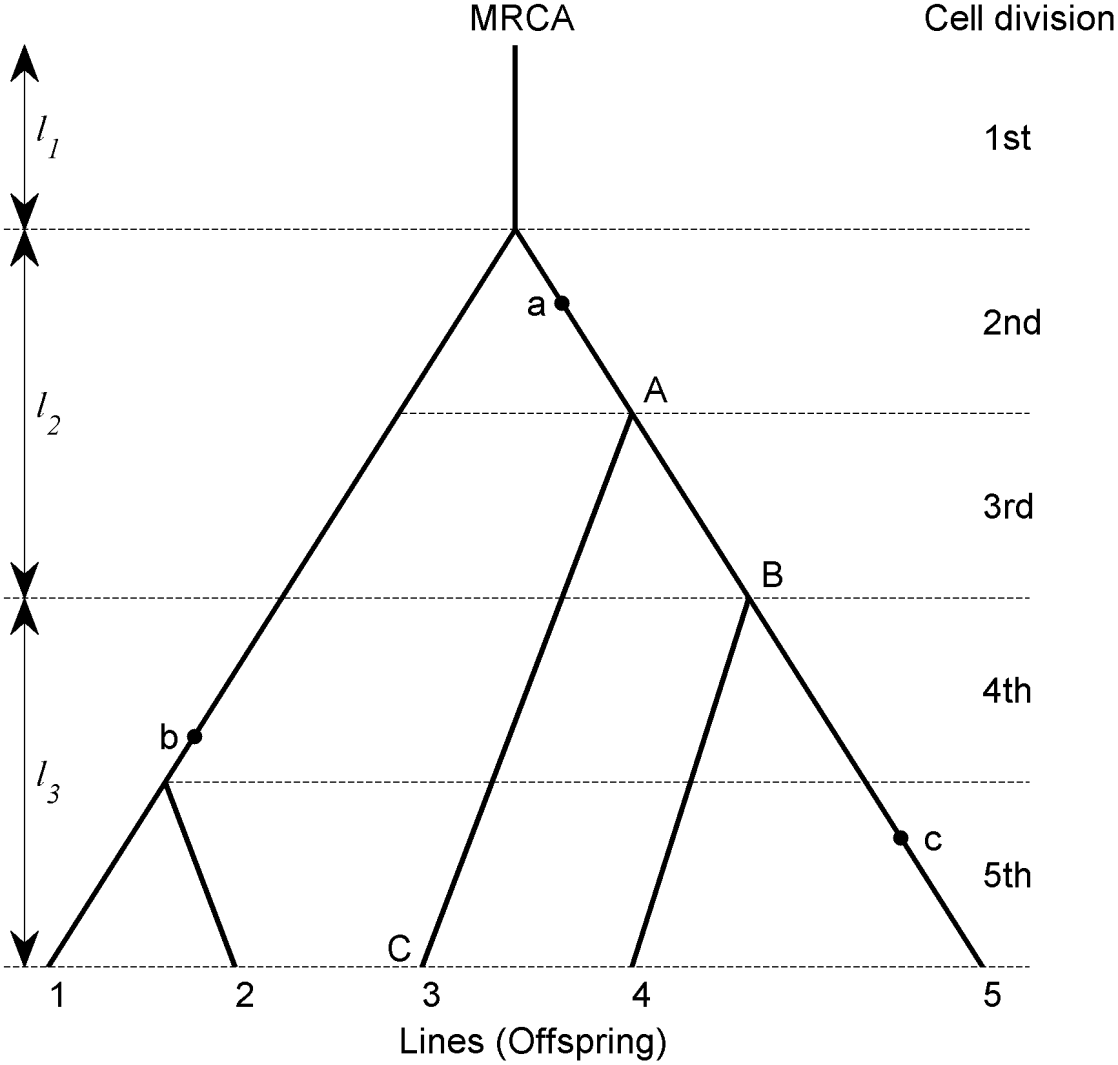
A genealogy of five lines. A family of male descendents (sperms) that come from an ancestor male *drosophila melanogaster* (ancestor sperm) can be represented as a genealogy. The numbers at the bottom of the Fig denote 5 lines (offspring or sample in terms of coalescent theory) of the family from the Most Recent Common Ancestor (MRCA), and the order of the numbers does not matter. Solid lines denote branches, filled dots denote mutations, and *l*_*j*_ s (*j* = 1,2,3) denote the number of cell divisions of a single line of the genealogy in interval *j* (combine coalescent times into fewer intervals because of the constraint of the computation burden, *l*_2_ and *l*_3_ includes two coalescent times each), and *l*_1_ = 1, *l*_2_ = 2, and *l*_3_ = 2. Branches can be classified according to the number of descendents in the sample. A branch is defined as a branch of size *i* if the branch has *i* descendents in the sample, for example, branch AB is a branch of size 2 and branch AC is a branch of size 1. A mutation is said to be a mutation of size *i* if the mutation occurred at the branch of size *i*, so the mutations a, b, and c are mutations of size 3, 2, and 1, respectively. Let *t*_*j*_ (*j* = 1,2,3) represents the number of cell divisions of all lines of the genealogy in interval *j*, so *t*_1_ = 1, *t*_2_ = 5, and *t*_3_ = 9, denote vector ***t*** ^*T*^ = (*t*_1_,*t*_2_,*t*_3_), ***t*** represents the total number of cell divisions in the genealogy.

For a sperm sample from a single male *Drosophila melanogaster*, their ancestral relationships lead to a sample genealogy that traces the common ancestor to the fertilized egg (the Most Recent Common Ancestor (MRCA)). Fig 1 gives an example of the genealogy of 5 sperms (which are often referred to as lines in genealogy).

Branches in a genealogy (i.e. the lines connecting different nodes in the genealogy) can be classified according to the number of descendents in the sample. A branch is said to be of size *i* if it has *i* lines (offspring) in the sample and a mutation is said to be of size *i* if it occurred at a branch of size *i*. Suppose the cell divisions from the fertilized egg to the germ cell are divided into *J* consecutive intervals, and the mutation rate is constant within each interval (see Fig 1). Furthermore, use the convention that the branch length corresponds to a certain number of cell divisions. Let *a*_*ij*_ be the number of cell divisions of all lines in the genealogy at the branch of size *i* in interval *J*, and vector i ***a***_*i*_ = (*a*_i1_,*a*_i2_,…,*a*_*ij*_)^*T*^, where the superscript *T* represents a transpose. ***a***_*i*_(*i* = 1,2,…,*n*) is the number of cell divisions of size *i* in the genealogy and *n* is the sample size of the genealogy. Additionally suppose that the mutation rate per cell division in interval *j* is *u*_*j*_(*j* = 1,2,…,*J*), define vector ***u*** = (*u*_1_,*u*_2_,…,*u*_*J*_)^*T*^. The overall mutation rate *μ* is defined as the sum of mutation rates over all divisions in the development. Thus given the estimate ${\hat{u}}_{j}$ of mutation rate *u*_*j*_, the estimate $\hat{\mu }$ of the overall mutation rate *μ* is

$$\hat{\mu }=\sum_{j=1}^{J}l_{j}{\hat{u}}_{j}$$

where *l*_*j*_ is the length of a single line in the interval *j* in the genealogy, that is, the number of cell divisions of a single line in interval *j* in the genealogy, see Fig 1.

For a genealogy, every cell division corresponds to a segment of a branch, assume *t*_*j*_(*j* = 1,2,…,*J*) is the number of cell divisions of all lines in the genealogy in interval *j*, see Fig 1. Define vector ***t*** = (*t*_1_,*t*_2_,…,*t*_*J*_)^*T*^, ***t*** is the total number of cell divisions (branch length) in the genealogy, $t=\sum_{i}a_{i}$. The number of mutations in a branch is commonly assumed to be a Poisson variable with the parameter equal to the product of the branch length and the mutation rate per cell division [7,8]. Therefore the number of mutations in a given genealogy is a Poisson variable with parameter ***t*** ^*T*^ ***u***. It follows that the probability that there is no mutation in a family is:

$$p_{0}=e^{-t^{T}u}.$$ (1)

From the experiment which yielded the data discussed here, mutations occurred in the subtree of a mutated branch will be masked [2] (Due to the fact that once a fly carried a recessive lethal mutation, further mutations will not change its status and thus were non-detectable in the experiments.) For example, the mutation c in Fig 1 will be masked by the mutation a. Fu [2] found that the probability for a mutation pattern <*i*_1_,…*i*_*l*_> in a genealogy is approximately

$$P(<i_{1},\cdots i_{l}>|g)\approx e^{-{\left[t-w(<i_{1},\cdots i_{l}>)\right]}^{T}u}S(<i_{1},\cdots i_{l}>,u)$$ (2)

where each element *i*_*k*_ in the mutation pattern <*i*_1_, *i*_*l*_> represents an identifiable mutation and its value is the number of offspring carrying the mutation. For example, the three mutations a, b, and c in Fig 1 can be denoted as the mutation pattern <3,2,1>. ***w***(<*i*_1_,…*i*_*l*_>) is the sum of branch lengths for branches that are descendants of those that harbor mutations. *S* is defined as

$$S(<i_{1},\cdots i_{l}>,u)=\sum_{l_{1}\cdots l_{m}}a_{l_{1}\cdots l_{m}}u_{l_{1}\cdots l_{m}}.$$

Since the genealogy for a given sample is usually unknown, we simulated a sufficient number of genealogies and calculated the mean value of the quantities of interest according to the typical application of the coalescent theory [1–3]. Replacing ***t***, ***w*** and ***a***_*i*_ by their corresponding mean values leads to an good approximation of the probability *P*(<*i*_1_,…*i*_*l*_>) which forms the basis for the maximum likelihood (ML) analysis [2].

One alternative method to the ML for parameter estimation is the minimum chi-square approach, which is to find parameter values that minimize chi-square like statistics. Berkson [12] considered five specific chi-square functions, including Pearson's $\chi_{P}^{2}=\sum \left[{\left(o-e\right)}^{2}/e\right]$ (*o* and *e* represent observed and expected frequencies, respectively). Neyman's $\chi_{N}^{2}=\sum \left[{\left(o-e\right)}^{2}/o\right]$, and log-likelihood $\chi_{\lambda }^{2}=2\sum o\text{ln}(o/e)$, and concluded that minimizing any of the chi-square statistics yielded an estimate asymptotically equivalent to the ML, while for smaller samples, the minimum chi-square method may even be superior in some circumstances. For computational simplicity, we consider two chi-square statistics in this paper: Pearson's $\chi_{P}^{2}$ and Neyman's $\chi_{N}^{2}$. (The following “chi-square” or notations “*χ* ^2^” with or without subscript in this paper are all referred to as the Neyman’s or (and) the Pearson’s “chi-square statistics or its value”, NOT “the chi-square distribution”!)

One can group terms according to their number of mutations as well as family size and uniformly define the Neyman’s and the Pearson’s chi-square as

$$X_{k}^{2}=\sum_{l=0}^{k}\sum_{n\geq 2}\sum_{<i_{1},\cdots i_{l}>}\frac{{\left(o_{n,<i_{1},\cdots ,i_{l}>}-e_{n,<i_{1},\cdots ,i_{l}>}\right)}^{2}}{x_{n,<i_{1},\cdots ,i_{l}>}}$$ (3)

where the inner summation is taken over all possible mutation patterns <*i*_1_,…*i*_*l*_> of length *l* for a given family size *n*. The *x* in the denominator of each term is either *o* or *e* and *k* is the maximum length of the observed mutation patterns. $X_{k}^{2}$ is the Neyman's $\chi_{N}^{2}$ if *x* = *o* and Pearson's $\chi_{P}^{2}$ if *x* = *e*. We will focus primarily on exploring the utility of $X_{1}^{2}$ (the mutation pattern length *k* = 1) which is a constrained weighted least square and can be solved easily.

We find that unless the mutation rate is very extreme, further simplification of Eq (2) is possible by ignoring the ***w***(<*i*_1_,…*i*_*l*_>) term without losing substantial accuracy, which leads to the probability that there is one exact mutation of size *i* in a family with family size *n*

$$P_{n}(<i>)\approx e^{-t^{T}u}({\left(a_{i}^{\left(n\right)}\right)}^{T}u)$$

where vector $a_{i}^{\left(n\right)}$ is the number of cell divisions of size *i* in a family with family size *n*. With the convention that <0> implies no mutation and ${\left(a_{0}^{\left(n\right)}\right)}^{T}u=1$, we have

$$X_{1}^{2}=\sum_{n\geq 2}\sum_{<i>}\frac{{\left(o_{n,<i>}-N_{n}e^{-t^{T}u}({\left(a_{i}^{\left(n\right)}\right)}^{T}u)\right)}^{2}}{x_{n,<i>}}$$ (4)

$$=\sum_{n\geq 2}(N_{n}e^{-t^{T}u})\sum_{i>0}\frac{{\left(o_{n,<i>}/(N_{n}e^{-t^{T}u})-{\left(a_{i}^{\left(n\right)}\right)}^{T}u\right)}^{2}}{x_{n,<i>}/(N_{n}e^{-t^{T}u})},$$ (5)

where *N*_*n*_ is the number of families with family size *n*. Since $N_{n}e^{-t^{T}u}$ is the expected number of families with family size *n* that does not have mutations, one approach is to replace this quantity by *o*_n,<0>_ which leads to

$$X_{1}^{2}=\sum_{n\geq 2}o_{n,<0>}\sum_{i>0}\frac{{\left(o_{n,<i>}/o_{n,<0>}-{\left(a_{i}^{\left(n\right)}\right)}^{T}u\right)}^{2}}{x_{n,<i>}/o_{n,<0>}}.$$ (6)

Since $o_{n,<i>}/o_{n,<0>}={\left(a_{i}^{\left(n\right)}\right)}^{T}u$ is a linear equation with respect to ***u***, it follows that the estimate of ***u*** by minimizing Neyman’s $\chi_{N,1}^{2}$ (*x*_*n*,*<i>*_ = *o*_*n*,*<i>*_ in Eq (6)) is a weighted least square estimate of ***u*** with the constraint ***u***≥0. This correspondence allows one to utilize existing theory, for example the Kuhn and Tucker theory [13,14] and algorithms for solving these quadratic programming problems, including the well-known Lemke complementary pivot algorithm [15–18], Active set method [19], Interior point method [20] and Newton method [21]. In this paper, we chose the Lemke complementary pivot algorithm [15–18] for its simplicity and wide implementation in computer packages, such as Mathematica [22]. For convenience of incorporating the search with other components of the analysis, we implemented the Lemke algorithm in Java. The correctness of the programming was assured by comparison of solutions from our Java program with those obtained from Mathematica.

The standard Lemke's algorithm can be applied for minimizing Neyman’s $\chi_{N,1}^{2}$ since the weights 1/(*o*_*n*,*<i>*_/*o*_*n*,*<o>*_) are constant before minimization, but the statistics are undefined when one or more *o* are zeros. Since the minimum non-zero observation is 1, one can replace zero *o*'s by 1.

For Pearson’s $\chi_{P,1}^{2}$ ($x_{n,<i>}=o_{n,<0>}{\left(a_{i}^{\left(n\right)}\right)}^{T}u$ in Eq (6)), minimization cannot be completed directly using the standard Lemke's algorithm because the inner weighting $1/({\left(a_{i}^{\left(n\right)}\right)}^{T}u)$ is dependent on the values of the unknown parameter ***u***. We propose to use the following iterative procedure:

1. Find an initial value for ***u*** satisfying the non-negative constraints.
2. Compute the numerical values of all the denominators in $\chi_{P,1}^{2}$ for the given initial value of ***u***.
3. Minimize $\chi_{P,1}^{2}$ by the Lemke algorithm treating the weights as constants and obtain a new estimate of ***u***.
4. Repeat steps 2–4 using the current ***u*** as the initial value of ***u*** until convergence is reached.

Since the purpose is to minimize Pearson's $\chi_{P,1}^{2}$, the iteration process stops when the minimum is clearly reached. In all the cases examined, including many thousands of simulated data set, the iteration procedure typically reduced the $\chi_{P,1}^{2}$ value monotonically to its minimum (or local minimum) in a few cycles and further iterations will lead to a steady state value which is often slightly larger than the minimum. To avoid being trapped in a local minimum, a number of different initial values can be tested. In our experience 5 well-spaced values for each dimension, thus a total of 5^*J*^ initial value sets, are sufficient to ensure finding the global minimum and the estimate that leads to the smallest $\chi_{P,1}^{2}$ is taken as the Pearson's estimate.

Furthermore, the $X_{1}^{2}$ in Eq (6) can be further simplified when we consider only a constant family size. For example, *n* = 20, $X_{1,1}^{2}=o_{20,<0>}\sum_{i>0}\frac{{\left(o_{20,<i>}/o_{20,<0>}-{\left(a_{i}^{\left(20\right)}\right)}^{T}u\right)}^{2}}{x_{20,<i>}/o_{20,<0>}}$, where the subscript “1,1” means one mutation pattern, one family size which can be used to analyze the data as reported in Gao et al. [1]. The corresponding Neyman’s and Pearson’s chi-square statistics for this one family size case are denoted as $\chi_{N,1,1}^{2}$ and $\chi_{P,1,1}^{2}$, respectively.

## Numerical Results

### Statistical properties of the $X_{1}^{2}$ estimator

Since the estimator does not have a simple analytical form, its statistical properties have to be evaluated numerically. As is common in statistical practice, bias and standard error are two primary measures of the properties of an estimator. We employed simulated samples for studying these properties, which can be obtained using the coalescent algorithm [1–3,7,8].

### One family size

We simulated several groups of mutation patterns for fixed family size (say, family size = 20). For each simulated sample under a given set of parameters, we applied the new method to obtain an estimate of the mutation rates. Since we consider only the families with mutations of no more than one in $X_{1,1}^{2}$ we discarded families with mutations greater than one. Therefore we only used part of the information if the sample includes families with mutations greater than one. The treatment of the families with mutations greater than one will be explained in the Discussion section.

The simulation can be separated into two independent steps according to the classical coalescent theory [7,8]. The first step is to simulate the genealogy and the second step is to superimpose mutations on the genealogy. We first simulated the coefficients ${\bar{a}}_{ij}$ s for 5 intervals and 38 cell divisions (Table 1) for fixed family size (*n* = 20) using the same method as in Gao et al. [1,3] and Fu [2]. Then we simulated mutation patterns using different initial mutation rates, and generated *Z* (for example, *Z* = 1000) cases for each initial mutation rate. We calculated *Z* estimators by minimizing Neyman's $\chi_{N,1,1}^{2}$ and Pearson's $\chi_{P,1,1}^{2}$, respectively using the Lemke algorithm for each group of simulations. To avoid the search procedure for Pearson's $\chi_{P,1,1}^{2}$ being trapped into a local optimal value, we tested a considerable number of initial values to obtain the global optimal estimation. Table 2 is the Neyman's estimation and the Pearson's optimal estimation for a considerably wide range of initial values, together with results from the ML method [1]. From the simulation results we can see that the estimation from minimization of the *χ* ^2^ (both Neyman’s and Pearson’s) and from the ML method are indeed asymptotically consistent. The estimate by minimizing Pearson's $\chi_{P,1,1}^{2}$ is slightly better than that of Neyman's in terms of unbiasedness because many observations are zero in the simulated mutation patterns.

**Table 1:** Simulated ${\bar{a}}_{ki}$, 20 lines per family, 5 intervals^a^, 2 million simulations, and 38 cell divisions.

*k*	${\bar{a}}_{k1}$	${\bar{a}}_{k2}$	${\bar{a}}_{k3}$	${\bar{a}}_{k4}$	${\bar{a}}_{k5}$
1	0.05028237	0.18482122	24.27570070	96.32421066	93.06592231
2	0.06784213	0.23011006	20.89096763	61.20499303	3.23522610
3	0.07823357	0.24062849	13.38313297	27.64166534	0.14672610
4	0.08673008	0.23875896	7.85545120	9.61194771	0.00563645
5	0.09415637	0.23191086	4.76238446	2.77231275	0.00017430
6	0.10076345	0.22031624	3.05434761	0.71554283	0.00000498
7	0.10566135	0.20469970	2.04687450	0.17854980	0.00000000
8	0.10885757	0.18783914	1.40841434	0.04467530	0.00000000
9	0.11161006	0.17002888	0.98125598	0.01066335	0.00000000
10	0.11211454	0.15119572	0.68993426	0.00270020	0.00000000
11	0.11161006	0.13237102	0.48290488	0.00067580	0.00000000
12	0.10885757	0.11362600	0.33614343	0.00012699	0.00000000
13	0.10566135	0.09733566	0.23387500	0.00003635	0.00000000
14	0.10076345	0.08119920	0.15908367	0.00001145	0.00000000
15	0.09415637	0.06621165	0.10514392	0.00000647	0.00000000
16	0.08673008	0.05296912	0.06897908	0.00000000	0.00000000
17	0.07823357	0.04101992	0.04241335	0.00000000	0.00000000
18	0.06784213	0.03062948	0.02500149	0.00000000	0.00000000
19	0.05028237	0.01984811	0.01326843	0.00000000	0.00000000
20	0.13980578	0.02652739	0.00949353	0.00000000	0.00000000

^a^ The 5 intervals are: [1,1], [2,2], [3,14], [15,33] and [34,38].

**Table 2:** Estimates of the mutation rate by minimizing Neyman's and Pearson’s *χ* ^2^ for 5 intervals (the intervals are the same as Table 1) and family size 20. The number of cases = 1000. (All the simulated data used in this paper has mask effect.).

Case	Method		*u*_1_	*u*_2_	*u*_*3*_	*u*_4_	*u*_5_
a	min^c^ $\chi_{N,1,1}^{2}$	mean	0.00035706	0.00036172	0.00056025	0.00061634	0.00058917
		std	0.00037065	0.00061824	0.00012403	0.00006603	0.00006398
		MSE	0.00000016	0.00000040	0.00000002	0.00000002	0.00000001
	min^c^ $\chi_{P,1,1}^{2}$	mean	0.00057439	0.00084619	0.00057393	0.00060853	0.00059269
		std	0.00056114	0.00095418	0.00013580	0.00006797	0.00006374
		MSE	0.00000032	0.00000103	0.00000002	0.00000002	0.00000001
	min^d^ $\chi_{N,1,1}^{2}$	mean	0.00037155	0.00034983	0.00048377	0.00051788	0.00049005
		std	0.00037983	0.00060305	0.00011947	0.00006156	0.00005939
		MSE	0.00000016	0.00000039	0.00000001	0.00000000	0.00000000
	min^d^ $\chi_{P,1,1}^{2}$	mean	0.00057937	0.00080615	0.00050008	0.00050922	0.00049384
		std	0.00055569	0.00090617	0.00012783	0.00006269	0.00005908
		MSE	0.00000032	0.00000091	0.00000002	0.00000000	0.00000000
	ML^e^	mean	0.00046238	0.00061622	0.00047761	0.00050787	0.00049595
		std	0.00039839	0.00052960	0.00009202	0.00005047	0.00005230
		MSE	0.00000016	0.00000029	0.00000001	0.00000000	0.00000000
b	min^c^ $\chi_{N,1,1}^{2}$	mean	0.00066175	0.00026745	0.00007761	0.00011884	0.00053327
		std	0.00039476	0.00042318	0.00005613	0.00002884	0.00003697
		MSE	0.00000027	0.00000021	0.00000000	0.00000000	0.00000000
	min^c^ $\chi_{P,1,1}^{2}$	mean	0.00088555	0.00051624	0.00008421	0.00011553	0.00053454
		std	0.00058761	0.00062980	0.00005852	0.00002904	0.00003678
		MSE	0.00000036	0.00000057	0.00000000	0.00000000	0.00000000
	min^d^ $\chi_{N,1,1}^{2}$	mean	0.00065994	0.00025198	0.00007253	0.00011103	0.00049531
		std	0.00038850	0.00041138	0.00005418	0.00002785	0.00003540
		MSE	0.00000027	0.00000019	0.00000000	0.00000000	0.00000000
	min^d^ $\chi_{P,1,1}^{2}$	mean	0.00088518	0.00048659	0.00007905	0.00010788	0.00049645
		std	0.00057854	0.00060725	0.00005675	0.00002826	0.00003524
		MSE	0.00000035	0.00000052	0.00000000	0.00000000	0.00000000
	ML^e^	mean	0.00080209	0.00038219	0.00008847	0.00010310	0.00049756
		std	0.00049468	0.00045320	0.00004117	0.00002249	0.00003426
		MSE	0.00000028	0.00000029	0.00000000	0.00000000	0.00000000

^a^ The true mutation rate u = [0.0005, 0.0005, 0.0005, 0.0005, 0.0005].

^b^ The true mutation rate u = [0.001, 0.0001, 0.0001, 0.0001, 0.0005].

^c^ Minimizing *χ* ^2^ by using all families and decomposing families with more than one mutation into several families. If the observed value is 0, replace it with 1 in Neyman's estimate.

^d^ Minimizing *χ* ^2^ by only using families with no more than one mutation. If the observed value is 0, replace it with 1 in Neyman's estimate.

^e^ Estimation using the maximum likelihood method as in Gao et al. [1].

Calculation of the minimizing *χ* ^2^ method is very rapid. For example, 1000 groups of estimates can be obtained in one to two seconds in the PC, while Gao et al.'s [1] ML method is much more time consuming.

### Multiple family sizes

We also simulated the varying family size situations similar to the configuration in Gao et al.'s [3] experimental data, the results are presented in Table 3. The mutation rate in the second interval has the largest bias and the mutation rate in the last 3 intervals fit the true mutation rate well. The minimization of Pearson's $\chi_{P,1}^{2}$ method is slightly better than that of Neyman's as a whole.

**Table 3:** Estimates of the mutation rate by minimizing Neyman's and Pearson's *χ* ^2^ for varying family size (from 2 to 35) and 5 intervals (the intervals are the same as Table 1). The number of cases = 2000.

Case	Method		*u*_1_	*u*_2_	*u*_*3*_	*u*_4_	*u*_5_
a	min^d^ $\chi_{N,1}^{2}$	mean	0.00005718	0.00050103	0.00005192	0.00008297	0.00009956
		std	0.00013024	0.00040519	0.00004820	0.00002207	0.00001609
		MSE	0.00000002	0.00000033	0.00000000	0.00000000	0.00000000
	min^d^ $\chi_{P,1}^{2}$	mean	0.00009090	0.00008922	0.00008901	0.00008911	0.00008906
		std	0.00014880	0.00014909	0.00014720	0.00014715	0.00014714
		MSE	0.00000002	0.00000002	0.00000002	0.00000002	0.00000002
b	min^d^ $\chi_{N,1}^{2}$	mean	0.00024827	0.00233125	0.00018928	0.00053522	0.00048934
		std	0.00046132	0.00126335	0.00013253	0.00005343	0.00003758
		MSE	0.00000028	0.00000495	0.00000011	0.00000000	0.00000000
	min^d^ $\chi_{P,1}^{2}$	mean	0.00060545	0.00078802	0.00051082	0.00050401	0.00049697
		std	0.00055825	0.00086289	0.00010808	0.00004779	0.00003650
		MSE	0.00000032	0.00000083	0.00000001	0.00000000	0.00000000
c	min^d^ $\chi_{N,1}^{2}$	mean	0.00098438	0.00361194	0.00050110	0.00109199	0.00096821
		std	0.00110979	0.00230323	0.00023313	0.00009138	0.00005871
		MSE	0.00000123	0.00001213	0.00000030	0.00000002	0.00000000
	min^d^ $\chi_{P,1}^{2}$	mean	0.00161448	0.00161003	0.00109328	0.00099999	0.00098870
		std	0.00111462	0.00176815	0.00019171	0.00008068	0.00005699
		MSE	0.00000162	0.00000350	0.00000005	0.00000001	0.00000000

^a^ The true mutation rate u = [0.0001, 0.0001, 0.0001, 0.0001, 0.0001].

^b^ The true mutation rate u = [0.0005, 0.0005, 0.0005, 0.0005, 0.0005].

^c^ The true mutation rate u = [0.001, 0.001, 0.001, 0.001, 0.001].

^d^ Minimizing *χ* ^2^ by only using families with zero and one mutation. If the observed value is 0, replace it with 1 in Neyman's estimate.

## Application to *Drosophila* data

### Family size equal to 20

We applied the minimum *χ* ^2^ method to the published data in Gao et al. [1]. The mutations used in our analysis were extracted from their Table 1 by considering only families with at most 1 mutation. Our estimation for the mutation rate by minimizing Neyman's and Pearson's *χ* ^2^ using the Lemke algorithm are respectively:

$${\hat{u}}_{N}^{T}=(0.00127011,0.00000000,0.00000000,0.00000000,0.00117405)$$

$${\hat{u}}_{P}^{T}=(0.00665943,0.00000000,0.00000000,0.00000000,0.00120312)$$

where the subscript *N* and *P* represent Neyman and Pearson, respectively. This result is consistent with the ML estimate ${\hat{u}}_{ML}^{T}$ = (0.005043, 0.000001, 0.000001, 0.000008, 0.001225) in Gao et al. [1]. The ML estimation used the families with at most two mutations, but the proportion of the families with two mutations is negligibly small, only 0.95%. The total mutation rates are respectively, ${\hat{\mu }}_{N}$ = 0.0071, ${\hat{\mu }}_{P}$ = 0.0127 (0.0125 in Gao et al. [1]). The Pearson's estimate is superior to that of Neyman's because there are several observations where mutations are zeros, which were replaced by 1s in Neyman's $\chi_{N,1,1}^{2}$, which caused certain bias. These results reconfirmed Gao et al.'s [1] conclusion about the varying mutation rate during the germline development in *Drosophila melanogaster*. For example, the mutation rate is the highest in the first interval, the second highest in the last interval, and the lowest in between these two intervals. Gao et al.'s [1] ML estimation is said to be nearly unbiased, this paper’s results are slightly different from Gao et al.'s [1], so our estimation is a bit biased.

### Family size varying from 2 to 35

By applying the minimizing *χ* ^2^ method to Gao et al.'s [3] whole data set, we can obtain the estimation of the mutation rate (Table 4). The Pearson's estimates slightly overestimated the mutation rate compared with that of the ML and the estimate from minimizing Pearson's $\chi_{P,1}^{2}$ is superior again. The tendencies are the same for both methods because they are asymptotically consistent.

**Table 4:** Estimates of the mutation rate for weighting all 34 family sizes (2–35) by using Gao et al.'s [3] data (The 6 intervals in Gao et al. [3] are: [1, 1], [2, 2], [3, 3], [4, 14], [15, 33], [34, 38].)

Lethality	Method	*u*_1_	*u*_2_	*u*_*3*_	*u*_4_	*u*_5_	*u*_6_	*μ*
[99%,100%)	min^a^ $\chi_{N,1}^{2}$	0.002488	0.000000	0.000000	0.000000	0.000000	0.000747	0.006225
	min^a^ $\chi_{P,1}^{2}$	0.006234	0.000000	0.000000	0.000000	0.000040	0.000802	0.010998
	ML^b^	0.004054	0.000000	0.000000	0.000001	0.000028	0.000850	0.008847
[98%,99%)	min^a^ $\chi_{N,1}^{2}$	0.011047	0.000000	0.000000	0.000000	0.000031	0.000500	0.014131
	min^a^ $\chi_{P,1}^{2}$	0.043618	0.000000	0.000000	0.000000	0.000034	0.000542	0.046974
	ML^b^	0.024460	0.000337	0.000006	0.000010	0.000067	0.000551	0.028941
[97%,98%)	min^a^ $\chi_{N,1}^{2}$	0.014709	0.000000	0.000000	0.000000	0.000052	0.000375	0.017568
	min^a^ $\chi_{P,1}^{2}$	0.078955	0.000000	0.000000	0.000000	0.000000	0.000424	0.081075
	ML^b^	0.039577	0.000605	0.000002	0.000002	0.000077	0.000398	0.043659
≥98%	min^a^ $\chi_{N,1}^{2}$	0.011186	0.000000	0.000000	0.000000	0.000026	0.001273	0.018038
	min^a^ $\chi_{P,1}^{2}$	0.051330	0.000000	0.000000	0.000000	0.000067	0.001355	0.059378
	ML^b^	0.028660	0.000272	0.000006	0.000018	0.000089	0.001443	0.038042
≥97%	min^a^ $\chi_{N,1}^{2}$	0.019509	0.000000	0.000000	0.000000	0.000046	0.001705	0.028916
	min^a^ $\chi_{P,1}^{2}$	0.151034	0.000000	0.000000	0.000000	0.000000	0.001900	0.160532
	ML^b^	0.067371	0.001258	0.000021	0.000031	0.000177	0.001954	0.082124

^a^ Minimizing *χ* ^2^ by only using families with zero and one mutation. If the observed value is 0, replace it with 1 for Neyman's estimate.

^b^ Gao et al.'s [3] Maximum Likelihood estimate.

## Conclusion and Discussion

A new method is developed in this paper for estimating the mutation rate during the germline development of male *Drosophila melanogaster* by minimizing the *χ* ^2^ statistics. This method is asymptotically equivalent to the maximum likelihood method with up to one mutation per family being considered and may be superior to the latter method for a small sample [12]. We considered two chi-squares in this paper: Pearson’s $\chi_{P}^{2}$ and Neyamn’s $\chi_{N}^{2}$. In fact, Neyman’s $\chi_{N}^{2}$ is more widely used than Pearson’s $\chi_{P}^{2}$ because the expected number of observations are often unknown. The largest advantage of the new method is its computational speed, which is achieved by the discovery that the *χ* ^2^ statistics can be simplified with approximation which turns the optimization problem into a quadratic programming problem, and there exists a global optimal value which can be found easily by Lemke’s algorithm. When we applied Neyman’s chi-square method to the experimental data, we often encounter the problem of division by zero which is caused by some zero observations. We circumvent the problem with two approaches. The first one is to replace the zero denominator by value 1 (since this is the minimum non-zero observation) and the second one is to rely on minimizing Pearson’s chi-square, which leads to an iterative procedure. We found that this second approach works well, and the estimator is slightly superior to the first approach.

We found that the estimate by minimizing Pearson's *χ* ^2^ is generally superior to that by minimizing Neyman's *χ* ^2^ unless the sample size is exceptionally large. If there are no zero observations (remember that it is required that there be at least one observation for every treatment in the goodness of fit test using chi-square) or that the number of zero observations is small, then the Neyman’s estimation will be superior by our simulation.

The estimate of the mutation rates using the new method is shown to be reasonably accurate based on simulations which had similar parameter configurations as Gao et al.’s [1, 3] and Fu's [2]. Applying the new method to the data in Gao et al. [1,3] yielded comparable estimates to those previously obtained, which enhances the confidence about the previous conclusions on the pattern of the variation of mutation rate during the germline development of male *Drosophila melanogaster*. The new estimation method can be used alone or together with other iterative methods (such as the ML) by supplying excellent initial parameter values. Therefore, the new method should be useful for future mutational analysis based on next-generation sequencing, which is gradually becoming the standard for studying within-individual polymorphism.

The main limitation of the new approach is that it only utilizes families with at most one mutation. This limitation mainly occurs because of the use of the constrained weighted least square method and quadratic Lemke algorithm. This is appropriate for the data we analyzed and is also suitable for sequence based studies for a relatively small region (such as a gene or a set of genes). When families with more than one mutation is frequent, the approach can still apply but it is becoming less desirable since only a fraction of the data is utilized. Nevertheless further extension to include families with more than one mutation will be needed to be more widely applicable.

There are several possible extensions of the methods and its application. In the real data, there are families that have more than one mutation, and ignoring those with more than one mutation is not ideal. One way to incorporate these types of families into the analysis is to pretend that each mutation had occurred in a separate family so that their pattern can be formally incorporated into the analysis. For example, a family with 2 mutations <2,1>, one mutation is the mutation of size 2, the other is of size 1. We can decompose this family into 2 families, one family is with a mutation of size 2, and the other family is with a mutation of size 1. This approach was found in our experience to be quite effective as long as the number of families with more than one mutation is not too large. For example, we used this decomposition method on the simulated data (see Table 2) and the real experimental data. When the mutation rate is small, the estimation is a little different compared to those only using families with mutations of zero and one. However, when the number of families with more than one mutation is large, the bias increases accordingly. We used this decomposition method on Gao et al.'s [1] published data. The estimates are, respectively,

$${\hat{u}}_{N}^{T}=(0.00258679,0.00000000,0.00000749,0.00000000,0.00137060)$$

$${\hat{u}}_{P}^{T}=(0.00786448,0.00000000,0.00000000,0.00000000,0.00139920)$$

The total mutation rates are respectively, ${\hat{\mu }}_{N}$ = 0.0095 and ${\hat{\mu }}_{P}$ = 0.0149 (0.0125 in Gao et al. [1]). These results are very similar to that of using the ML method in Gao et al. [1] because the proportion of families with more than one mutation accounts for only a small fraction of available data. For example, the number of families with 0, 1, and 2 mutations are 7664, 872, and 82, respectively [1]. In the total 8,618 families, the families with two mutations accounts for only 0.95% (= 82/8618) of all families and ${\hat{u}}_{P}^{T}$ is again superior to ${\hat{u}}_{N}^{T}$.

Since the new method is highly efficient computationally, we were able to explore finer divisions of the developmental stage of male *Drosophila melanogaster*. Such levels of exploration would have been very difficult if not entirely impossible using the previous Maximum Likelihood approaches. Our minimizing *χ* ^2^ method can deal quickly with more intervals.

One reason there are small differences between the two estimates from minimizing the *χ* ^2^ method and the ML method is that the two methods are asymptotically consistent [12]. In practical usage we often can not arrive at the limitation. Secondly, in order to take advantage of the rapid speed of the Lemke algorithm we discard families with more than one mutation or decompose the family with more than one mutations into several families whose number of mutations is exactly one in each family. However, both approaches may lead to some errors. Thirdly, the treatment of the denominator in Neyman's *χ* ^2^ when the denominator is zero also affects the estimate. In general, according to our simulation, the larger the sample size, the more mutation patterns become available, so the smaller the bias of the estimation and the estimation by minimizing Neyman's *χ* ^2^ is becoming superior to that of Pearson's. Conversely the estimation by minimizing Pearson's *χ* ^2^ is more superior for small sample size. Lastly, we did not take the mask effect into consideration thus far in our model. The estimation for the unmasked mutation is little different from that of the masked mutation by our simulation, and the estimate for unmasked mutation seems to be slightly superior.

The newly developed methods work most effectively when there are at most only one mutation in a given family. This limitation does not pose a serious issue when such methods are applied in the future to polymorphism data generated by next-generation sequencing techniques. This is because a vast majority of such polymorphic sites will likely come from different regions which are revealed by short sequence reads from overlapping but nevertheless different sample of cells, which effectively create many families (genealogies) in which the vast majority contain at most one mutation.
